# Observations on the Ergot of Rye

**Published:** 1826-01

**Authors:** 

**Affiliations:** Surgeon, Bristol.


### ART. VII.—*Observations on the Ergot of Rye.* By — CLARK, Esq. Surgeon, Bristol.

ON looking over the continental Journals a few years since, my attention was frequently arrested by the effects described as resulting from the Secale cornutum, and I anticipated the value which such a remedy would prove to our Pharmacopœia. Not having any opportunity myself of making a trial of its efficacy, I trusted to the accouuts of those whose testimony, from their experience and advantages, I considered as satisfactory. Their report, however, was very unfavourable, and I had nearly given up the idea of the drug proving any acquisition, till, through the medium of this Journal, such cases were brought before the public as could not but attract considerable attention, and claim the patient investigation of every reader: I allude more particularly to the interesting details of Dr. H. DAVIES. I feel pleasure in adding, that I am now enabled in some measure to corroborate his statements, by the result of the following cases.

CASE I.—Mrs. S—, a strong athletic woman, thirty-eight years of age, (third labour,) was delivered of a fine child about eight o'clock A.M. half an hour before I reached her residence. The attendants were anxiously waiting the removal of the after-birth. On examining the abdomen, it was found very hard and tumid, and by attention I thought I could discover the situation of a second infant. No presentation, however, could be ascertained by an examination per vaginam. The woman, though in good spirits, and free from fatigue, had no pains till noon, and those very slight; but, during their continuance, I was enabled to ascertain that the head of another child presented. No alteration occurred till four P.M.; the woman still very easy. The pelvis being very capacious, the external parts freely dilated by the passage of the first infant, and nothing but some slight pains wanting to complete the labour, I ordered a scruple of the secale cornutum in tea. Precisely twelve minutes after its exhibition, the woman was seized with a pain, which, though not very violent, continued from three to five minutes, and caused the child to descend into the pelvis, so that I could clearly ascertain the whole extent of the head and direction of the sutures. This was succeeded by others; and they continued, with some little intermission, for nearly two hours, when the second child was expelled; and, in less than fifteen minutes, was followed by two placentæ. The mother and children did exceedingly well.

Case II.—Mrs. C—, æt. thirty-eight, (second child.) Labour commenced about six o'clock Saturday morning, October 22d. Had been ill three hours before I saw her. The membranes were ruptured, and the waters driven off with considerable violence. On making an examination, the os uteri was found but little dilated; the head presenting naturally, but high at the anterior and superior aperture of the pelvis. The pains continued strong, and at very short intervals, during the whole of the day, and increased towards night: still the labour proceeded slowly.

About nine on Sunday morning, the os uteri was nearly effaced: the head made a perceptible descent. I consequently anticipated a speedy termination of the labour: but in this I was disappointed, by the cessation of any efficient pains: they were frequent and sharp, wearying, without propelling in the slightest degree. At noon, I gave a scruple of the secale cornutum; and, in less than fifteen minutes, it was followed by a pain of greater force and bearing than any she had experienced during the labour: they remitted but little till the child was born, which happened about an hour and a half after its exhibition.

Case III.—Mrs. Bullock, a short stout woman, had been in labour three days before I visited her, which was late on Tuesday night, the 8th. On making an examination, the external parts were soft, well lubricated with mucus, and disposed to yield; the os uteri dilated about one inch in diameter; the head lying high at the brim of the pelvis; the efforts of the uterus frequent and tiresome. Under these circumstances, I hoped, if efficient pains came on, the labour would terminate favourably. I directed the midwife to give an enema, and promised to call in the morning.

Wednesday, ten A.M.—Things much the same; the os uteri, however, more dilated and loose; the woman very anxious. I now ordered a scruple of the powdered secale cornutum in tea, which produced, in ten minutes, a bearing down pain, that lasted, without intermission, for seven or eight minutes. The woman was standing, and, from the violence of the effort, I almost thought the child would have been expelled: others succeeded, but not so forcibly, for two hours. The pains now remitted, and I gave a second dose of the secale. The propelling efforts of the uterus again returned; the vectis was applied, and the child delivered, about an hour and a quarter after the exhibition of the last dose. The mother well; child dead.

It may be well to remark here, that this same woman informed me that her last labour was exceedingly protracted and alarming: the child then also still-born.

Though these cases are but few, they are the only ones in which I have given the ergot of rye a trial, and the result in each has been uniform. No unpleasant symptoms attended its exhibition; the pulse in none of the cases was in the least accelerated. Sickness attended one; but nausea had been complained of previously to its administration. The only alteration perceptible was the increase or rise of bearing efficient pains, within a few minutes after its being taken. From these circumstances, I think I may safely infer, with Dr. Davies, that its action is peculiarly directed to the uterus; and that, under proper management, the dose being sufficient, and the quality of the medicine good, its effects will be found as well marked and decided as that of any other class of medicines with which we are acquainted.

